# Hydrophobic Amino Acid Content in Onions as Potential Fingerprints of Geographical Origin: The Case of *Rossa da Inverno sel. Rojo Duro*

**DOI:** 10.3390/molecules23061259

**Published:** 2018-05-25

**Authors:** Federica Ianni, Antonella Lisanti, Maura Marinozzi, Emidio Camaioni, Lucia Pucciarini, Andrea Massoli, Roccaldo Sardella, Luciano Concezzi, Benedetto Natalini

**Affiliations:** 1 Department of Pharmaceutical Sciences, Section of Chemistry and Technology of Drugs, University of Perugia, Via del Liceo 1, 06123 Perugia, Italy; federica.ianni@chimfarm.unipg.it (F.I.); antonellalisanti86@gmail.com (A.L.); maura.marinozzi@unipg.it (M.M.); emidio.camaioni@unipg.it (E.C.); lucia.pucciarini@hotmail.it (L.P.); benedetto.natalini@unipg.it (B.N.); 2 3A-Umbria Agrifood Technology Park, Fraz. Pantalla, 06059 Todi, Italy; reteagrometeo@parco3a.org (A.M.); lconcezzi@parco3a.org (L.C.)

**Keywords:** *Rossa da inverno sel. Rojo Duro* onion *cultivar*, geographical origin, amino acids content, HPLC analysis, statistical evaluations, food traceability

## Abstract

In this study, we were interested in comparing the amino acid profile in a specific variety of onion, *Rossa da inverno sel. Rojo Duro*, produced in two different Italian sites: the Cannara (Umbria region) and Imola (Emilia Romagna region) sites. Onions were cultivated in a comparable manner, mostly in terms of the mineral fertilization, seeding, and harvesting stages, as well as good weed control. Furthermore, in both regions, the plants were irrigated by the water sprinkler method and subjected to similar temperature and weather conditions. A further group of Cannara onions that were grown by micro-irrigation was also evaluated. After the extraction of the free amino acid mixture, an ion-pairing reversed-phase high performance liquid chromatography-evaporative light scattering detector (IP-RP HPLC-ELSD) method allowed for the separation and detection of almost all the standard proteinogenic amino acids. However, only the peaks corresponding to leucine (Leu), phenylalanine (Phe), and tryptophan (Trp), were present in all the investigated samples and they were unaffected from the matrix interfering peaks. The use of the beeswarm/box plots revealed that the content of Leu and Phe were markedly influenced by the geographical origin of the onions (with *** *p* << 0.001 for Phe), but not by the irrigation procedure. The applied HPLC method was validated in terms of the specificity, the linearity (a logarithm transformation was applied for the method linearization), the limit of detection (LOD) and limit of quantification (LOQ), the accuracy (≥90% for inter-day Recovery percentage), and the precision (≤10.51 for the inter-day RSD percentage), before the quantitative assay of Leu, Phe, and Trp in the onion samples. These preliminary findings are a good starting point for considering the quantity of the specific amino acids in the *Rossa da inverno sel. Rojo Duro* variety as a fingerprint of its geographical origin.

## 1. Introduction

Onions (*Allium cepa* L.) are the second most used vegetable worldwide after tomatoes [1]. A continuous interest is directed to the selection of the varieties and to the production of fresh and processed products with defined organoleptic and healthy properties. Onions are a valuable source of phenolic substances, especially quercetin and its glycosides, sulphur compounds, phenolic acids, vitamins and minerals, while a limited content of amino acids is present. Nevertheless, specific amino acids have a well-established and important role in the protein turnover and transamination processes in onions [2], while the presence of “umami” amino acids (that is, glutamic acid) was found to influence the sensory response and the characteristic taste associated to the vegetable [2,3].

We have long been interested in the study and definition of the properties of onions from Cannara, a small town in the Umbria region (Italy) [4,5,6]. In particular, in the frame of a broader project, we were interested, inter alia, at comparing the amino acid content in a specific variety of onion (*Rossa da inverno sel. Rojo Duro*) produced in two different locations: Cannara (group A) and Imola (Emilia Romagna region, Italy, group B). In both places, the onions were cultivated and harvested in the same way, and irrigated by water sprinkler method.

The amino acid content was appraised by using an ion-pairing reversed-phase high performance liquid chromatography-evaporative light scattering detector (IP-RP HPLC-ELSD) methodology. A further group of Cannara onions (group C) grown using water micro-irrigation, was also taken into account in the setting of the study. 

The role of amino acid analysis in food chemistry is well-recognized, not only to assess the product biological value, but also as a characterization parameter of different food sources [7,8,9,10]. 

In general, as far as botanical species are concerned, the evaluation of the composition of specific metabolites could be used as a criterion to evaluate the proceedings of the production of a particular variety, pointing out a plausible relationship with the growing location, the soil, and the weather conditions [11,12]. Accordingly, the appraisal of the type and levels of these metabolites could provide useful information about the variability in terms of organoleptic and nutritional properties [13,14,15].

During the study, we observed that the levels of the amino acids leucine (Leu), phenylalanine (Phe), and tryptophan (Trp) were different between the samples from Cannara and Imola. Accordingly, in the present work, we tried to assess a relationship between the content of these amino acids and the geographical origin of the onion cultivar, thus, contributing to favor the food traceability.

## 2. Results and Discussion

**Figure 1:**
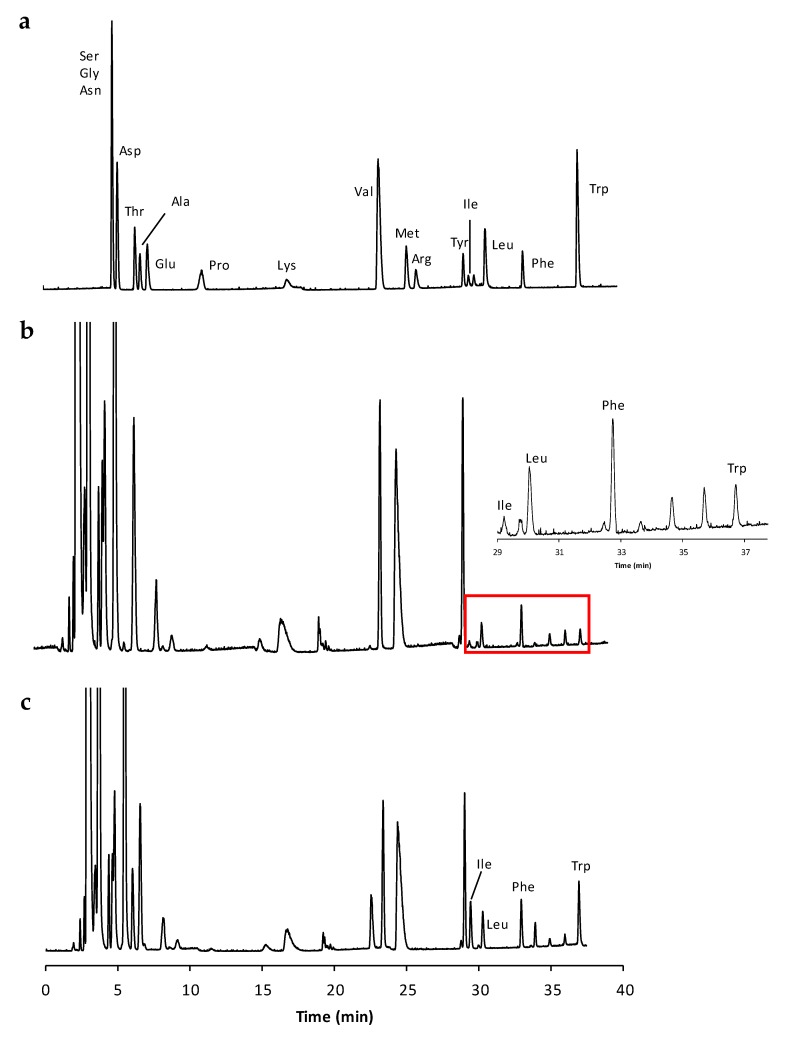
The chromatogram of (**a**) a standard amino acid mixture; (**b**) an extracted sample; and (**c**) a real sample spiked with a standard amino acid mixture. The enlarged section of the chromatogram in the time-window containing the three amino acids Leu, Phe, and Trp is highlighted. The Y-axis is in the mV scale.

The amino acid pool in the lyophilized samples was extracted with deionized water according to the procedure described in Section 3.5. The amino acid profile was then determined by applying an IP-RP HPLC method developed by our group in the frame of a previous study [16] focused on the analysis of cheese extracts. The already established chromatographic method is based on the use of heptafluorobutyric acid (HFBA) as an IP reagent, which offers the advantage to increase the analyte lipophilicity, its retention into an RP setting and, hence, the quality of the chromatographic performance in terms of selectivity and efficiency. By relying upon a non-polar end-capped RP-18 column and a 7.0 mM HFBA concentration in the aqueous eluent component (see Section 3.2 and Section 3.6 for details), the previously optimized gradient program is able to produce a profitable direct separation of many underivatized proteinogenic amino acids as readily evident from Figure 1a. Instead, the exemplary chromatogram of a real onion extract is shown in Figure 1b. 

On the basis of the comparison between the retention times of the peaks in each analyzed extract with those of a standard amino acid mixture, the following amino acids were identified in almost all the analyzed extracts: threonine (Thr), alanine (Ala), glutamic acid (Glu), valine (Val), arginine (Arg), isoleucine (Ile), leucine (Leu), phenylalanine (Phe), and tryptophan (Trp). Unfortunately, during the analysis of the many extracts, the co-elution of some of the above amino acids with the unidentified matrix deriving peaks occurred. Only the peaks corresponding to the three amino acids Leu, Phe, and Trp, were found in all the investigated samples and fully resolved from the other peaks in the chromatogram. Although we are aware that amino acids other than Leu, Phe, Trp are more abundant in onions [2,17,18], in the present study, the focus was exclusively given to these compounds for the reason explained above. These compounds were also considered for further analyses and quantifications.

The confirmation of their chemical identity was further apprised with the use of a triple quadrupole mass spectrometry (MS) detector equipped with an ESI source, by applying a similar IP-RP HPLC method developed by other authors [19] (the data are not shown). Indeed, the volatility of HFBA makes it highly compatible to LC-MS applications.

As clearly evident from Figure 1a, the peaks of isoleucine and leucine are very well separated and the possibility to distinguish the two species is maintained in the real sample. The chromatogram of the real sample with spiked isoleucine, leucine, phenylalanine, and tryptophan is shown in Figure 1c. 

From the literature data [2,20], it is possible to assess that isobaric species (ions) other than leucine and isoleucine are absent in onions. On this basis, we deem the species attribution made through the LC-MS analysis as realistic.

The amino acid analysis is often carried out after dedicated derivatization procedures aimed at introducing hydrophobic labels on the molecular structure [21,22,23,24]. Several derivatization reagents and procedures have been proposed so far and most of them suffer from many of the relevant drawbacks typically accompanying indirect analyses: the generation of interfering by-products; different derivatization rate for distinct amino acids, the non-quantitative recovery of the purification step, and so forth. Based on the above assumptions, direct methods of analysis should be the elective choice, especially when quantitative assays in rather complex matrices are required. The direct HPLC method applied in the present study was revealed to be particularly suited for the analysis of hydrophobic amino acids, as a consequence of the peculiar matrices under investigation. Indeed, the selected procedure utilized to extract the amino acidic component from the vegetable tissue, non-specifically enriched the extract of other polar constituents (the group of peaks in the first part of the chromatogram in Figure 1b) without compromising the chromatographic selectivity for the more retained hydrophobic compounds. 

### 2.1. Method Validation and Amino Acid Quantification 

As stated above, the HPLC method applied for amino acid analysis was developed and optimized in a previous study [16], while it has been validated here for a reliable quantitation of Leu, Phe, and Trp.

The content of the selected amino acids in the extract samples was determined by using the external calibration method, by correlating the logarithm peak area versus the logarithm analyte concentration values [25]. Usually, when an ELSD is used, a non-linear (almost always exponential) relationship between the output signal (area value, A) and the corresponding analyte concentrations (m) occurs (Equation (1)) when a wide range of concentrations is considered [26,27,28].

(1) $$A={\mathrm{am}}^{b}$$

**Figure 2:**
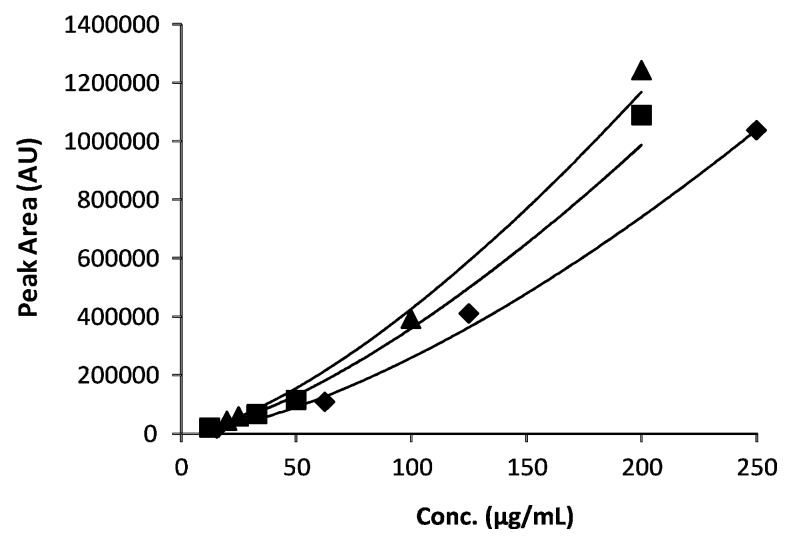
The calibration curves obtained for the three selected amino acids (◆ Leu; ■ Phe, ▲ Trp).

In all these cases, the logarithm transformation is the common way to linearize the exponential profile of area versus the concentration value plots (Figure 2). 

By employing the general Equation (2), the three calibration curves were thus obtained in the present study where the concentration ranges spanned over one order of magnitude was considered. All three calibration curves were characterized by appreciably high R^2^ values (Table 1).

(2) log A=b log m+log a

The regression equations reported in Table 1 were used to validate the chromatographic method and for quantitative analyses. Appreciably low LOD and LOQ values were calculated for the investigated amino acids. The method was also validated for precision and accuracy, in both the short- (intra-day) and the long-term (inter-day) periods. 

As reported in Table 2, a very profitable precision of the method was diagnosed in the short period. Accordingly, a comparable and low range of variation of the RSD % values (from 0.53 up to 9.5%) was observed during the consecutive three days of analysis, thus, ensuring a profitable stability of our analytical method. In accordance with this outcome, the acceptable RSD % values (ranging from 4.71 to 10.51%) were also recorded when the long-term (inter-day) precision was evaluated (Table 3). 

The rather high RSD % value that sometimes turned out could be tentatively ascribed to the so-called “instrumental fatigue” [29]. A decline in the output stability after prolonged use is a rather common situation with ELSDs. However, these values did not compromise the statistical quality of the method to an unacceptable extent for the purpose of the study.

The percentage of the recovery, the so-called “Recovery test” [30], was employed to estimate the accuracy of the ion-pairing reversed-phase high performance liquid chromatography-evaporative light scattering detector (IP-RP HPLC-ELSD) method. As reported in Table 2 and Table 3, the acceptable percentages of recovery were obtained: in the case of the intra-day analyses ranging from 84.08 up to 118.20 (Table 2), whereas during long-term runs from 90.49 to 105.16 (Table 3). 

The excellent results achieved in the validation step prompted us to apply the HPLC method for the content determination of the selected amino acids in an extended set of onion samples (groups A–C, see Section 3.3 and Section 3.4 for details). 

Based on the regression equations in Table 1, the average concentrations of the three amino acids were calculated and the data were shown in Table 4. Even though the determined concentration values for the three amino acids lay within rather narrow ranges, we preferred to use the logarithmically linearized curves instead of the linear portion of the exponential profiles. Indeed, as readily evident from the plots in Figure 2, a different degree of linearity characterizes the three curves in the vicinity of the estimated concentration values. 

The large standard deviation values can be tentatively ascribed to the wide variability in the bulb weight (see Section 3.5 for details) which might have some effect in the amino acid content and, to a lesser extent, in the extraction process.

As clearly evident from the data in Table 4, the concentrations of three amino acids from the group B (samples from Imola) are greater than those found in the other two groups (A and C: onion samples collected in Cannara). However, being amino acids in small amounts in onions, these differences should not, in principle, have a significant health impact. 

### 2.2. Statistical Evaluation

In order to highlight differences in the content of Leu, Phe, and Trp in the samples with different geographical origins (groups A and B), a further and deeper statistical evaluation was performed.

Many known plots are available and used to show distributions of univariate data. Tukey introduced the box and whiskers plot as part of his toolkit for exploratory data analysis [31]. These are particularly useful for comparing distributions across groups when other statistical methods such as analysis of variance (ANOVA) and Tukey Honestly Significant Difference (HSD) tests are employed. Furthermore, to visualize the data points on the box plot representation, a beeswarm plot was also implemented. Indeed, the superimposition of both plots is useful to gain a very rich description of the underlying distribution. 

**Figure 3:**
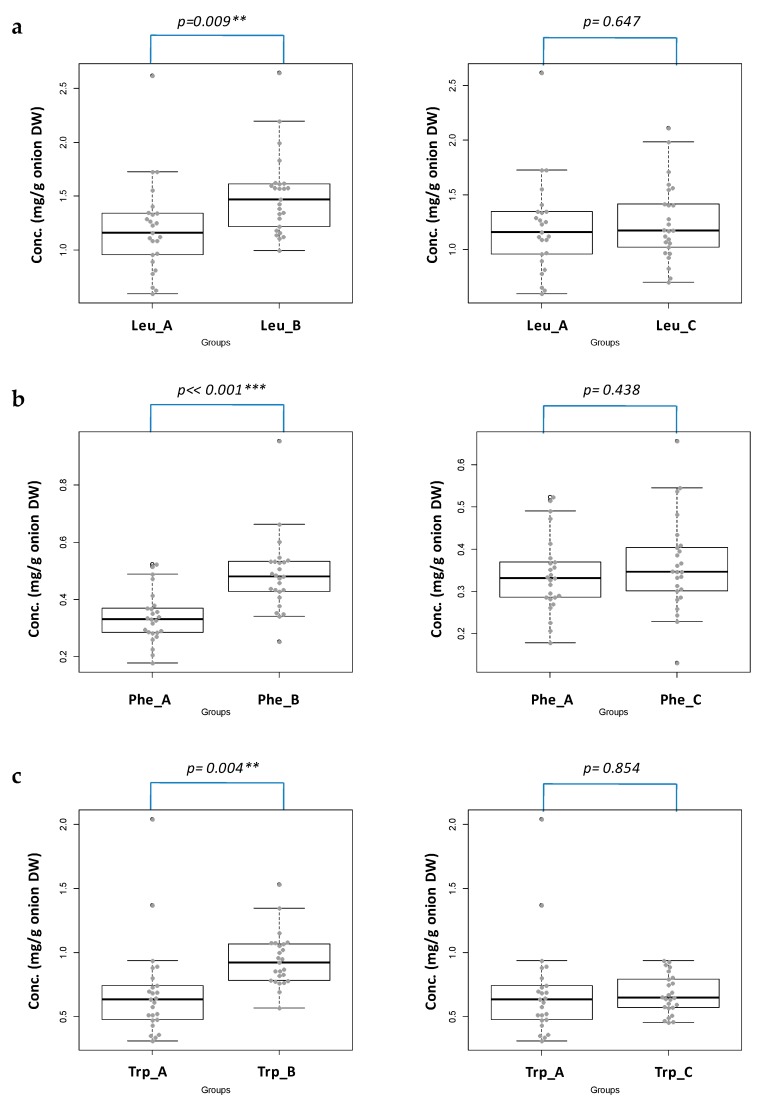
The Beeswarm/box plots with ANOVA/Tukey HSD analyses of the Leu (**a**); Phe (**b**); and Trp (**c**) content on the three sampled groups (left panels A versus B; right panels A versus C).

By following this statistical approach, the obtained data relative to the Leu, Phe, and Trp content, were extrapolated in such a way and the results were depicted in Figure 3.

The difference of the amino acid content between groups A and B is statistically significant. Indeed, the content level values of Leu and Trp from the onions cultivated in Cannara compared with those produced in Imola are significant (group A vs. B, ** *p* = 0.009 and 0.004, respectively). In the case of Phe, the differences between group A versus B are even more significant (*** *p* << 0.001). Therefore, as a matter of fact, the geographical origin can influence the content level values of Leu, Phe, and Trp in a statistically significant way.

From Figure 3, it is also clear that the irrigation mode does not affect the content of the selected amino acids: the difference in the content of the three selected amino acids are, indeed, not statistically significant (*p* >> 0.05). This last part of the study strongly suggests a geographically-related content of the species under investigation.

## 3. Materials and Methods

### 3.1. Reagents

Pure water for the HPLC analyses was obtained from a New Human Power I Scholar (Human Corporation, Seoul, Korea) purification system. All standard amino acids, as well as the eluent component acetonitrile (MeCN) and the ion-pair reagent heptafluorobutyric acid (HFBA), were of analytical grade and purchased from Sigma-Aldrich (Milan, Italy).

### 3.2. Instrumentation

The HPLC analyses were carried out on a Shimadzu (Kyoto, Japan) Class Prominence equipped with two LC 20 AD pumps, an SPD M20A photodiode array detector, a CBM 20A system controller, and a Rheodyne 7725i injector (Rheodyne, Cotati, CA, USA) with a 20 μL stainless steel loop. 

A Varian 385-LC evaporative light scattering detector (ELSD) (Agilent Technologies, Santa Clara, CA, USA) was utilized for the HPLC analyses. The analog-to-digital conversion of the output signal from the ELSD was allowed by a common interface device. The adopted operative ELSD conditions for the analysis were a 50 °C nebulization temperature, a 70 °C evaporation temperature, a 2 L/min auxiliary gas flow rate (air), and 1 as the gain factor.

A Prevail C-18 (Phenomenex, Torrance, CA, USA), 250 mm × 4.6 mm i.d., 5 μm, was used as the analytical column. The column was conditioned with the selected mobile phase at a 1.0 mL/min flow rate for at least 40 min before use. All the analyses were carried out at a 1.0 mL/min flow rate. The column temperature was kept at 25 °C with a Grace (Sedriano, Italy) heather/chiller (Model 7956R) thermostat. 

The Centrifuge Rotina 380 (Hettich, Tuttlingen, Germany) was employed for the extraction of amino acids from the freeze-dried onion samples.

### 3.3. Onion Sources

Group A: onion samples cultivated in Cannara (Province of Perugia, Umbria Region, Italy) and irrigated by water sprinkler method.

Group B: onion samples cultivated in Imola (Province of Bologna, Emilia Romagna Region, Italy) and irrigated by water sprinkler method.

Group C: onion samples cultivated in Cannara (Province of Perugia, Umbria Region, Italy) and irrigated by the micro-irrigation method.

All samples were provided by local farmers association, able to certify the cultivation characteristics and modalities. All onion samples were managed and sampled by the 3A-Parco Tecnologico Agroalimentare dell’Umbria Società Consortile a r.l. (Todi, Italy). 

### 3.4. Soil Sampling and Treatment

Both in Cannara and Imola, the onions were cultivated in a comparable manner, mostly in terms of the mineral fertilization, seeding, and harvesting stage, as well as the weed control. Furthermore, in both regions, the plants were irrigated by the water sprinkler method and subjected to similar temperature and weather conditions. Details on the cultivation characteristics and modalities are summarized in Table 5. 

In Cannara (groups A and C), the cultivation soil was divided into eight parcels. Four of them were irrigated by water sprinkler method through the use of oscillating sprinklers, while the remaining four zones were submitted to drip-spray micro-irrigation by the use of dynamic self-compensating micro-sprinklers. 

### 3.5. Sample Preparation and Extraction of Free Amino Acids

For each of the three groups A–C of onion *Rossa da inverno sel. Rojo Duro*, 25 bulbs were selected and each bulb was individually managed. Accordingly, each bulb was deprived of the outer drier, weighed, and chopped. The obtained mixture was subsequently freeze-dried and stored at 4 °C in sealed vials. The average weight of the fresh onions was about 122.30 g (±30.05), the corresponding mean weight value of the freeze-dried (DW) samples was 13.67 g (±3.63). 

The extraction of the amino acidic component from each of the previously obtained freeze-dried material from the 75 bulbs (that is, 25 per group) was performed according to a protocol described in the literature [32] with some modifications. In particular, 20 mL of distilled water was added to 1.0 g of freeze-dried onion sample. The obtained suspension was maintained under magnetic stirring for 3 min at 0 °C (ice bath) and centrifuged at 10,000 rpm for 15 min. This operation was consecutively repeated three times by re-suspending the pellet every time.

The final solution containing the amino acidic component was filtered under a vacuum through a 0.45 µm nylon filter. Each obtained solution was lyophilized again and stored at 4 °C in sealed vials until use. Each of the 75 extracts was analyzed in triplicate. 

### 3.6. Amino Acid Separation and Quantitation

Each extract was analyzed by using a previously developed IP-RP HPLC-ELSD methodology [16]. Samples were prepared at a concentration of 25 mg/mL, filtered through a nylon 0.45 µm filter and analyzed in triplicate.

The mobile phase gradient was obtained from eluent A (7 mM HFBA in pure water) and eluent B (net MeCN) as follows: 0–10 min 100% A, 10–30 min from 100 up to 75% A, 30–38 min from 75 up to 70% A, 38–39 min 100% A, 39–70 min 100% A.

### 3.7. Method Validation 

The amino acid content in the onion samples was determined using a chromatographic external calibration method. For each of three amino acids of interest (Leu, Phe, Trp), four calibration solutions were prepared and run in triplicate. The average of the corresponding peak area values was employed to build-up the regression line.

The method was validated in terms of specificity, linearity, accuracy, precision, and limit of detection (LOD) and Limit of quantification (LOQ). The precision and accuracy were estimated in both the short- (intra-day) and the long-term (inter-day) period. 

#### 3.7.1. Selectivity

Very appreciable separation (α) and resolution factor (R_S_) values between the peaks of the three amino acids Leu, Phe, and Trp were achieved in the selected experimental conditions. Moreover, no interference peaks were identified within the investigated analysis time.

#### 3.7.2. Linearity

For each of the three amino acids of interest, calibration curves obtained after the logarithm transformation of the peak area and concentration values were used.

The log-log curves were always obtained with high R^2^ and suitably used to appraise LOD and LOQ, as well as the precision and accuracy of the method (Table 1).

#### 3.7.3. LOD and LOQ

The LOD and LOQ values were calculated according to the following equations (Equations (3) and (4)):

(3) $$C_{\mathrm{LOD}}=3.3\frac{\sigma_{y}}{b}$$

(4) $$C_{\mathrm{LOQ}}=10\frac{\sigma_{y}}{b}$$

where C_LOD_ and C_LOQ_ are the sample concentrations corresponding to the LOD and LOQ, respectively, σ_y_ is the standard error of the corresponding regression, and b is the slope of the relative calibration equation (Table 1).

#### 3.7.4. Intra-Day and Inter-Day Precision and Accuracy

The method was validated for precision and accuracy, in both the short- (intra-day) and the long-term (inter-day) period. 

The intra-day precision was assessed for each of the three investigated amino acids with the equations listed in Table 1. For all compounds, an external set of two control solutions, with a concentration as indicated in Table 2, was run in triplicate. The procedure was repeated for a period of three consecutive days. The previously estimated mathematical models (Table 1) were then used to calculate the concentrations of the control solutions (observed concentrations, Table 2). The intra-day precision was evaluated as the relative standard deviation (RSD %) among the concentration values achieved from consecutive injections. For each control solution, the variation within the replicate injections performed during a three-consecutive day period and, hence, a total of nine injections, was used to calculate the inter-day precision (Table 3). 

The percentage of the recovery, the so-called “Recovery test” [30] was employed as a test to estimate the accuracy of our IP-RP HPLC-ELSD method. 

Similarly, for the estimation of short and long-term precision, the intra-day and inter-day accuracies were also determined with the same external solutions. Accordingly, while the former was determined by taking into account the three replicated runs for each control solution within a single day (Table 2), for the latter, the average value from nine determinations, along three days of analysis, was considered (Table 3). 

### 3.8. Statistical Methods

The boxplot and statistical analyses were performed with the aid of the open source software CRAN-R version 3.3.0. (http://www.R-project.org) [33]. In a classical boxplot, the horizontal line within the box indicates the median, the boundaries of the box indicate the 25th- and 75th-percentile, the whiskers indicate the highest and lowest values of the results, and the outliers are displayed as circles. In the present study, the box plot representation was overlaid with a beeswarm plot. 

A beeswarm plot is a 2D visualization technique where the experimental data points are plotted relative to a fixed reference axis without the overlapping of the data points. It is useful to display the measured values for each data point and also the relative distribution of these values.

One-way ANOVA (Analysis of Variance) was used as a statistical test to assess the differences in the means between the groups. Tukey’s HSD (Honest Significant Difference) methodology, at the confidence level of 95%, was further employed for multiple comparisons between all pair-wise means to determine how they differ [31]. The *p* < 0.01 (**) values were considered statistically significant.

## 4. Conclusions

In the present study, the chromatographic analysis of the hydrophobic amino acids Leu, Phe, and Trp in the *Rossa da inverno sel. Rojo Duro* onion cultivar farmed in a comparable manner in Cannara (Umbria Region, Italy) and in Imola (Emilia Romagna Region, Italy), was carried out by applying an IP-RP HPLC-ELSD method developed in the setting of a previous study.

The high quality of the method, validated in terms of specificity, linearity, LOD and LOQ, accuracy, and precision was demonstrated and was revealed to be useful for the quantification of the three selected amino acids in the onion samples. 

The statistical evaluation, based on the combination of the box plot representation with the beeswarm plot, indicated that the content of amino acids Leu, Phe, Trp was not affected by the irrigation mode, but was clearly and significantly influenced by the geographical origin of the onions (Cannara vs. Imola). 

Although further studies are needed to fully rationalize our results, these preliminary findings can represent a good starting point for considering the quantity of these specific amino acids in the *Rossa da inverno sel. Rojo Duro* onion cultivar as a fingerprint of its geographical origin. Moreover, the developed approach can be applied to other onion cultivars/varieties, thus, contributing to their characterization and traceability. The results achieved in the present work could represent the basis of a new and additional way to characterize vegetable foods.

## Figures and Tables

**Table 1:** The calibration data for the selected amino acids (AAs): regression equations, correlation coefficient (R^2^) values, explored linearity ranges, and LOD and LOQ values.

AA	Regression Eq.	R^2^	Linearity Conc. Range (µg/mL)	LOD (µg/mL)	LOQ (µg/mL)
Leu	y = 1.52(±0.07)x + 2.38(±0.14)	0.9951	15.6–250	0.15	0.44
Phe	y = 1.45(±0.08)x + 2.65(±0.144)	0.9940	12.5–200	1.44	2.95
Trp	y = 1.45(±0.04)x + 2.72(±0.07)	0.9984	25–200	0.08	0.23

**Table 2:** The statistical analysis of the three selected amino acids in the short period (intra-day precision and accuracy values).

AA	Solution #	Day	Theoretical Conc. (µg/mL)	Mean Observed Conc. (µg/mL)	n ^a^	Precision (RSD %)	Accuracy (Recovery %)
Leu	1	1	31.20	27.89	3	1.05	89.38
		2		28.64		9.50	91.81
		3		31.09		3.82	99.66
	2	1	160.00	161.54	3	2.83	100.96
		2		150.92		5.80	94.32
		3		171.65		4.69	107.28
Phe	1	1	25.00	21.02	3	3.75	84.08
		2		23.52		6.36	94.07
		3		23.55		2.12	94.22
	2	1	100.00	118.20	3	0.53	118.20
		2		100.79		9.12	100.79
		3		96.49		2.83	96.49
Trp	1	1	33.00	28.43	3	3.35	86.15
		2		28.87		2.81	87.48
		3		32.29		4.15	97.84
	2	1	143.00	135.64	3	4.90	94.85
		2		137.36		2.62	96.06
		3		145.90		3.41	102.03

^a^ Number of replicates.

**Table 3:** The statistical analysis of the three selected amino acids in the long period (inter-day precision and accuracy values).

AA	Solution #	Theoretical Conc. (µg/mL)	Mean Observed Conc. (µg/mL)	n ^a^	Precision (RSD %)	Accuracy (Recovery %)
Leu	1	31.20	29.21	9	7.13	93.61
	2	160.00	163.94		6.72	102.46
Phe	1	25.00	22.70	9	6.77	90.79
	2	100.00	105.16		10.51	105.16
Trp	1	33.00	29.86	9	6.86	90.49
	2	143.00	139.63		4.71	97.65

^a^ Number of replicates.

**Table 4:** The means ± SEM of concentration values determined for the selected amino acids of interest in the three groups studied (A–C). SEM is for “standard error of the mean”.

Group	Mean Conc. ± SEM (mg/g Onion DW ^a^ ± SEM)		
	Leu	Phe	Trp
A	37.4 ± 13.4 (1.197 ± 0.428)	10.6 ± 2.7 (0.339 ± 0.090)	20.0 ± 7.4 (0.690 ± 0.366)
B	49.9 ± 13.5 (1.504 ± 0.373)	16.3 ± 5.0 (0.486 ± 0.133)	31.4 ± 6.6 (0.945 ± 0.209)
C	41.1 ± 11.3 (1.250 ± 0.357)	11.9 ± 3.6 (0.360 ± 0.111)	22.2 ± 4.6 (0.680 ± 0.152)

^a^ DW = dried-weight.

**Table 5:** Cultivation characteristics and modalities of the onion samples with both irrigation methods.

Preliminary stage	Soil digging (25–30 cm)
Complementary processes	Soil harrowing
Depth mineral fertilization	50 kg/ha N-25 kg/ha P_2_O_5_-60 kg/ha K_2_O
	TIMAC Agro-Timasprint-0.5 t/ha
	Organic-mineral fertilizer
	NPK (CaO-MgO-SO_3_) with Boron (B)
	10-5-12 (8-2-24) + 0.1 + 7.5 C
Seeding stage	Distance between rows = 14 cm
	Distance between plant on the same row = 4 cm
	(Seeding density ~178 seeds/m^2^) (~4.0 kg/ha)
Surface mineral fertilization	39 kg/ha N
	0.15 t/ha NH_4_NO_3_
Phytosanitary measures	Metalaxil-m-Ridmil Gold^®^ (*Peronospora Schleideni*)
	Pyrimethanil-Scala^®^ (*Botrytis squamosa*, *Botrytis allii*, *Botrytis cinerea*)
Weed control	Mechanical or manual control
Harvesting stage	September 2013, first week
